# Supplementary material for: Striatal hub of dynamic and stabilized prediction coding in forebrain networks for olfactory reinforcement learning
Source: Nat Commun. 2022 Jun 8;13:3305. doi: 10.1038/s41467-022-30978-1 (PMC9177857; doi:10.1038/s41467-022-30978-1)
Supplement: Supplementary file 1 — Supplementary Information [file 41467_2022_30978_MOESM1_ESM.pdf]

Supplementary Information for

Striatal hub of dynamic and stabilized prediction coding in forebrain networks for olfactory reinforcement learning

Winkelmeier et al.

Includes:

Supplementary Figures 1-10

Supplementary Table 1

SUPPLEMENTARY FIGURES

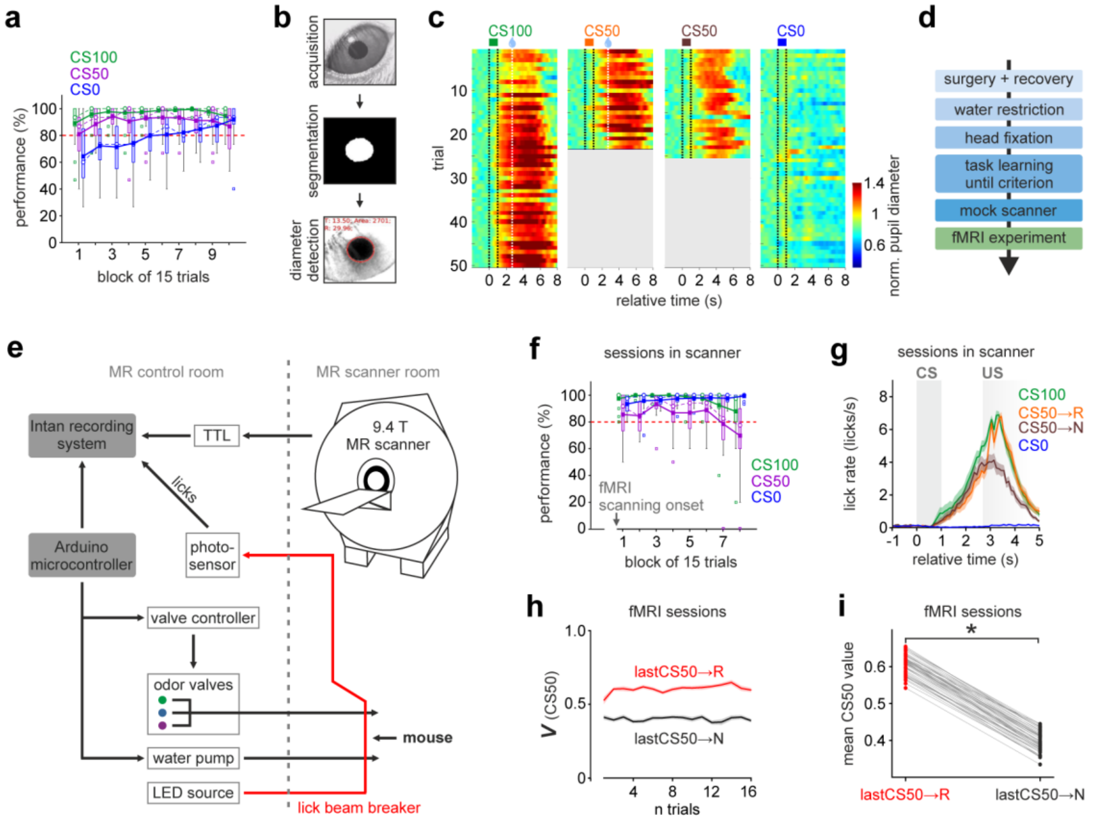

Supplementary Figure 1. Trained mice display differential anticipatory responses to olfactory stimulus-outcome pairs and dynamic value update by recent outcome-history. Related to Figure 1 and 2.

a, Performance curves for the three CS types in the mouse cohort shown in Fig. 1. The data contain the last three training sessions before fMRI (n=69 session from 23 mice). Performance curves discriminate learning of no-go (all CS0) and go trials (all CS50 and CS100). The performance criterion was met for no-go trials if animals licked less than 3 times in total in the anticipatory and reward window, or at least three times for the go trials (see Methods for detailed information). One trial block had 5 trials of each type. Mice performed consistently above criterion. Solid lines indicate average performance while dashed lines indicate median performance.

b, Pupil image processing. Conversion from raw video to binary frames for pupil segmentation and pupil diameter detection.

c, Pupil responses to each trial type from one example session. Color indicates an increase (red) or decrease (blue) from baseline.

d, Standard procedure with surgery and training before the fMRI experiments.

e, Schematic of the MRI-compatible setup. Odors were delivered through a custom-built olfactometer. Water was delivered by a remote syringe pump. Licks were detected with custom-built optics. The setup was controlled by Arduino microcontrollers.

f-g, Performance curves (f) and average lick rates ± SEM (g) for the sessions during fMRI acquisition (n=51 session from 18 mice).

h, Same as Fig. 2d but computed with fMRI CS-US sequences. A dynamic update of reward prediction in CS50 trials by the prior cue-specific outcome was also reflected in the TD modeling of the fMRI cohort. Since mice already performed 20-30 trials before fMRI acquisition started and reached a stable performance (f), initial value conditions were set to the reward probabilities associated with each CS.

i, Average CS50 values from TD model for lastCS50→R and lastCS50→N (one data point per session) were significantly different depending on the outcome of the previous CS50 trial (two-sided paired Wilcoxon signed rank, $Z = 6.2$; $p = 5.2$ e-10, n=51 session from 18 mice).

In the figure: * indicates $p < 0.05$ (see Supplementary Table 1 for exact p-values and test details). Box plots: The bounds of the box represent 25th to 75th percentiles. The center indicates the median. The lower and upper whiskers represent the minimum and maximum values, excluding outliers. Source data are provided as a Source Data file.

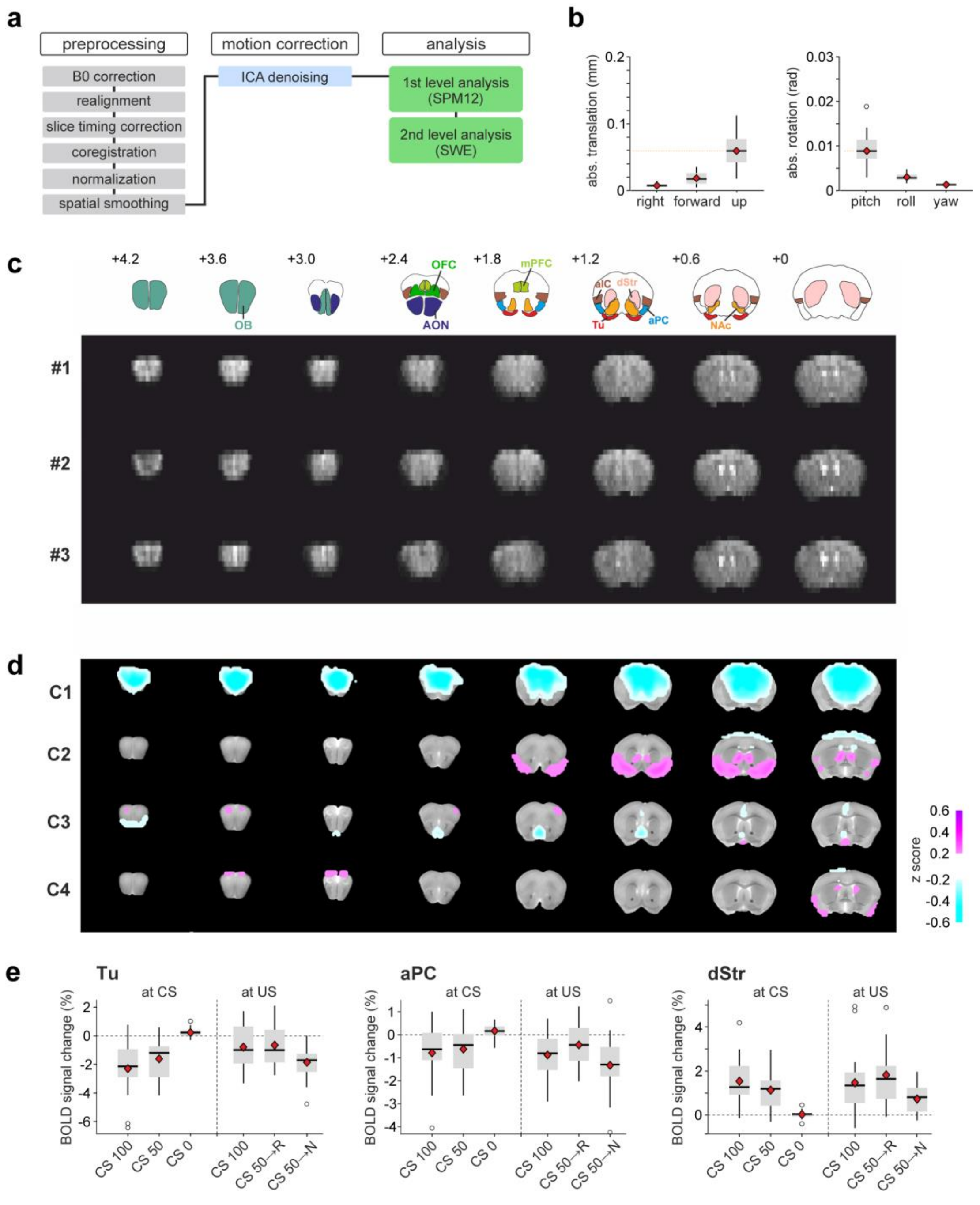

Supplementary Figure 2. Awake fMRI during task performance. Related to Figure 3.
a, Processing pipeline of fMRI data. See Methods for detailed information.
b, Session-averaged absolute realignment parameters for (left) the three translations and (right) the three rotations (n = 51 sessions in 18 animals). Realignment parameters are extracted from the fMRI preprocessing and serve as quantification of head motion after habituation to the scanner environment. Red rhombuses indicate the mean across sessions.
c, Top: Anatomical illustration of olfactory, striatal and higher-order regions (location from Bregma indicated in mm). Abbreviations: anterior olfactory nucleus (AON), anterior piriform cortex (aPC), dorsal striatum (dStr), agranular insular cortex (aIC), olfactory bulb (OB), medial prefrontal cortex (mPFC), nucleus accumbens (Nac), olfactory tubercle (Tu) and orbitofrontal cortex (OFC). Bottom: Examples of functional images in 8 coronal slices for three different animals. Displayed images are after initial preprocessing (realignment, field map distortion correction, slice-timing correction, spatial normalization), but without smoothing or motion artifact correction. For display purposes, images are masked with a template brain mask.**d,** Z-statistical maps showing the four motion-related independent components of the group ICA. Motion-related components were removed before first level analysis was conducted.
e, Magnitude of BOLD responses, expressed as percent signal change values (PSC) averaged over all sessions (n = 51) for Tu and aPC. Additionally, the dorsal striatum is shown as an exemplary region with widespread positive BOLD response in the paradigm. As detailed in the Methods section, we scaled PSC values in a region-specific manner, to avoid underestimating PSC in ventral regions, which have lower absolute signal intensities owing to the dorsally-located surface receive coil. Red rhombuses indicate mean PSC.
Box plots in the figure: The bounds of the box represent 25th to 75th percentiles. The center indicates the median. The lower and upper whiskers represent the minimum and maximum values, excluding outliers. Source data are provided as a Source Data file.

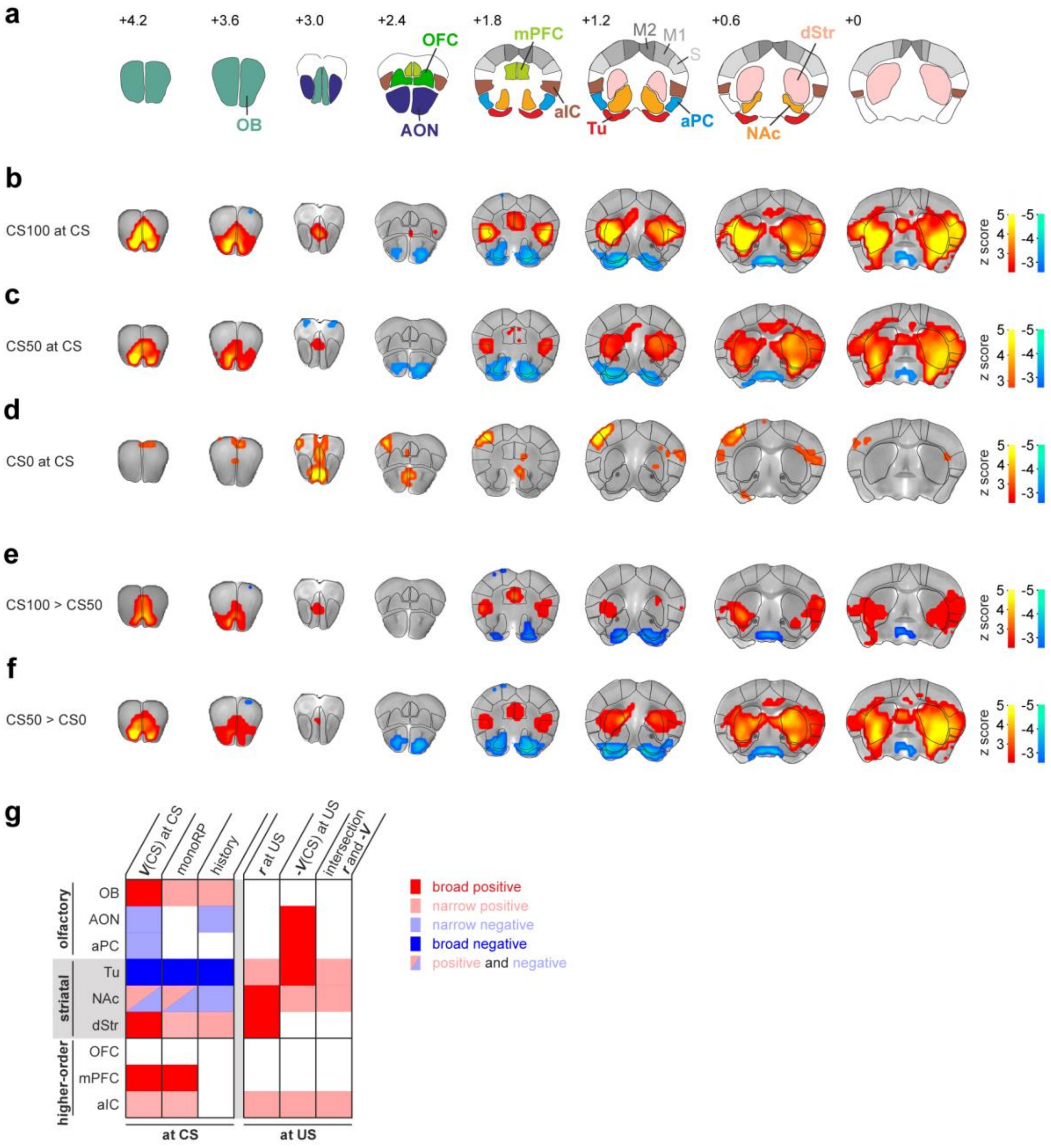

Supplementary Figure 3. Olfactory association-learning network encodes monotonic RP and prediction error components. Related to Figure 3.

a, Anatomical illustration of olfactory, striatal and higher-order regions (location from Bregma indicated in mm).

b-d, Group-level Z-statistical maps for the (b) CS100, (c) CS50, and (d) CS0 regressors (n = 51 sessions in 18 animals). Statistical threshold was set to $p < 0.025$ false discovery rate (FDR)-corrected, for two-sided testing (as for the other maps unless otherwise indicated). Red colors indicate areas activated by the respective CS events, while blue colors indicate deactivation. Olfactory and striatal areas were primarily recruited.

e, Group-level Z-statistical contrast maps for CS100>CS50 used for the RP intersection. The analysis was restricted to the regions associated with $V(CS)$ from Fig. 3c.

f, Same as (e) showing the contrast maps for CS50>CS0.

g, Schematic representation of BOLD correlates in defined ROIs for the analyses from Fig 3c-h as described in the Results section. Red color spectrum indicates ROIs with positive correlation, while blue color indicates negative correlation. Color intensity qualitatively reflects the broadness of responses.

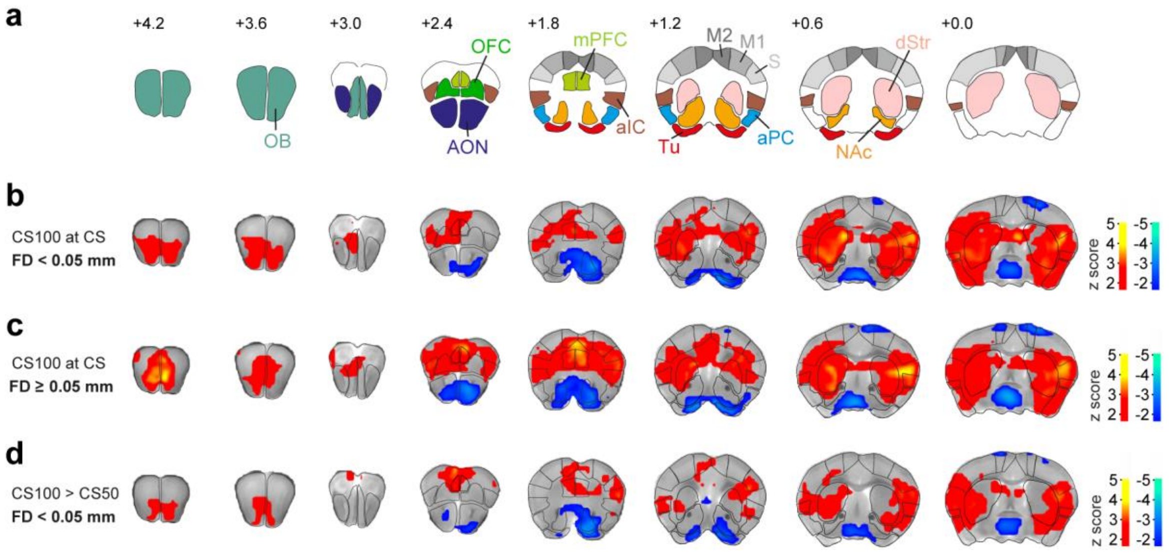

Supplementary Figure 4. BOLD response patterns at CS are robust to motion. Related to Figure 3.
a, Anatomical illustration of olfactory, striatal and higher-order regions (location from Bregma indicated in mm).
b-c, The pattern of BOLD responses was similar when considering only trials with a maximum frame-wise displacement (FD) of 0.05 mm in the two image frames at and following CS onset (low-motion trials), and trials with an FD above this cut-off (high-motion trials). For this analysis, only sessions were included that had, per CS type, at least 10 low-motion trials, yielding a balanced average number of low- and high-motion trials for CS50 and CS100 in 16 sessions. The CS100 events in high- and low-motion trials were modeled by separate regressors, replacing the CS100 odor regressor in GLM 2; the same was applied to CS50. To illustrate the anatomical pattern of BOLD responses in this subsample, statistical threshold was set to $p<0.05$ uncorrected, with minimum cluster size $k = 10$. Note that this lenient statistical threshold does not allow for inferences per se, but for comparing anatomical patterns of activation/deactivation between strata. This is shown for the response to CS100 in (b) low-motion and (c) high-motion trials.
d, Value differentiation as reflected in the contrast between CS100 and CS50 including only low-motion events ($p<0.05$ uncorrected, cluster size $k = 10$) shows a similar pattern as in the original analysis (see Supplementary Fig. 3e). Together these results support that the BOLD response patterns associated with value coding are not an artifact due to motion.

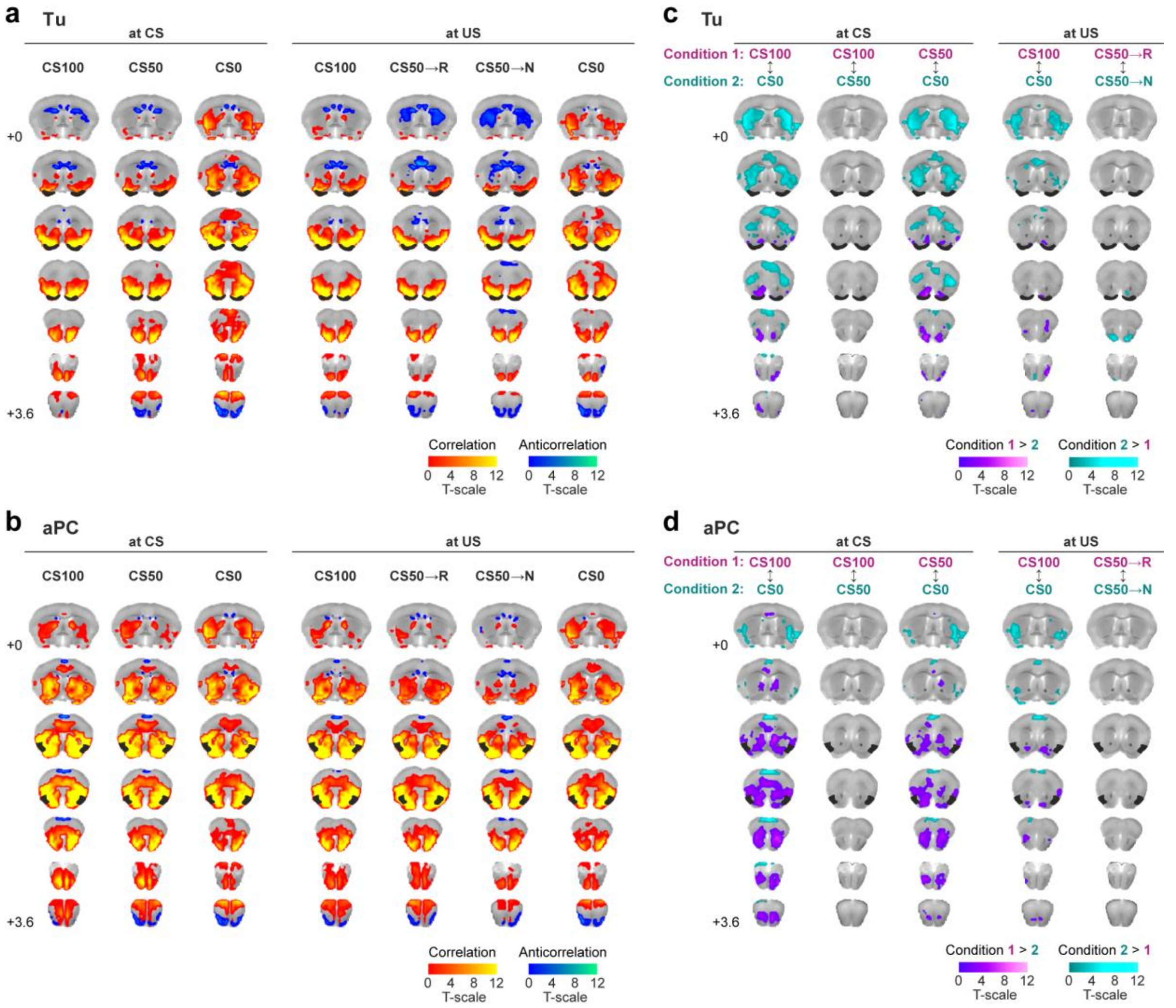

Supplementary Figure 5. Learned odor-reward associations recruit differential networks functionally connected to Tu and aPC. Related to Figure 4.

a, Group-level beta-series correlation maps showing seed-based functional connectivity for CS and US for each trial type in Tu (n = 51 sessions in 18 animals). Statistical threshold was set to $p < 0.025$, false discovery rate (FDR)-corrected, for two- sided testing (as for the other maps). Red color spectrum indicates a positive correlation, while blue color indicates anticorrelation. A composite mask of all ROIs listed in Fig. 4a was created and used for this analysis: olfactory bulb, anterior olfactory nucleus, aPC, as well as Tu, Nac, dorsal striatum, orbitofrontal cortex, medial prefrontal cortex and agranular insular cortex. Some regions, like the olfactory bulb, exhibited both correlation and anti-correlation with the Tu seed region.

b, Same as (a) but with aPC as seed region.

c, Group-level beta-series correlation maps showing different cross-trial type contrasts for seed-based analysis in Tu. The seed ROI is schematically illustrated (black).

d, Same as (c) but with aPC as seed region.

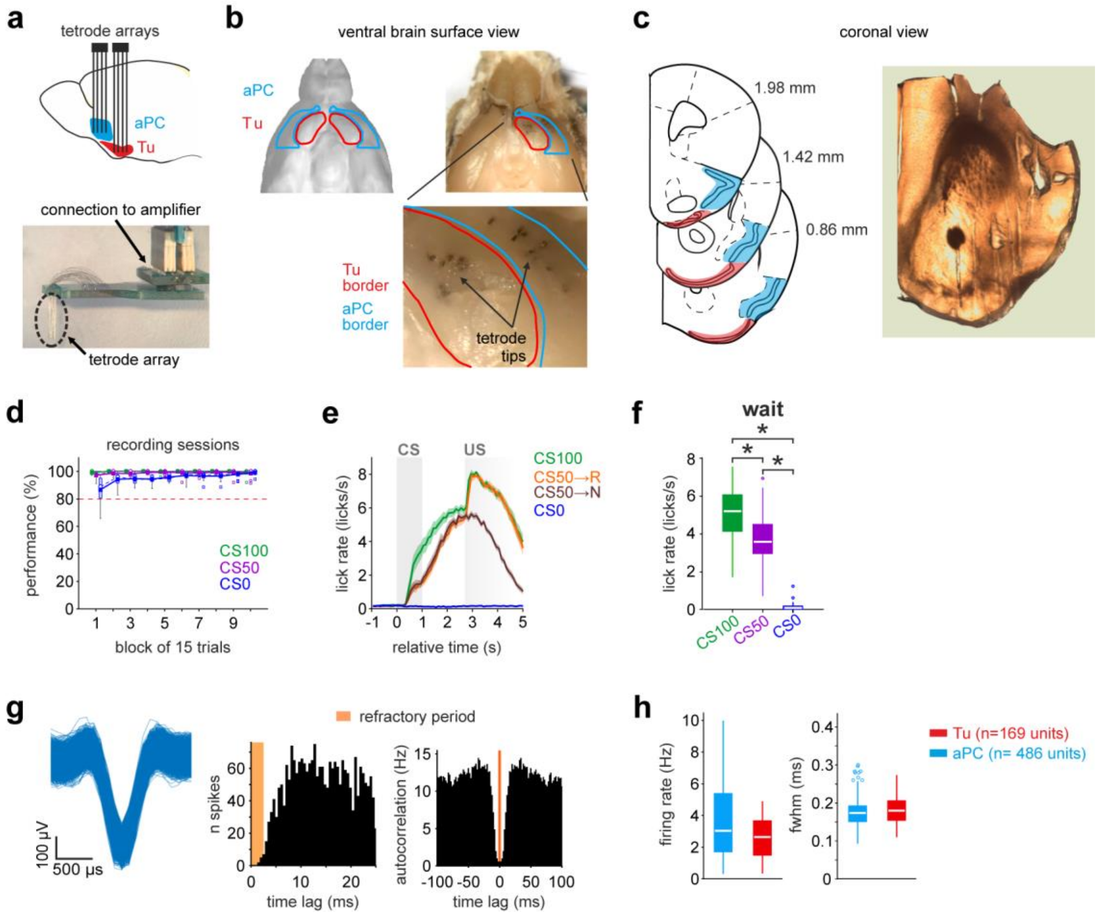

Supplementary Figure 6. Single-unit recordings in the olfactory tubercle and the anterior piriform cortex.
Related to Figure 5.
a, Scheme with the anatomical relation between Tu and aPC in sagittal view (top) and an example of a tetrode array connected to the breakout board of the head stage connector (bottom). Tetrode arrays were implanted unilaterally.
b, Ventral forebrain view of the mouse brain including ROI definitions. The tetrodes tips are visible in the Tu and aPC on the ventral surface of the brain.
c, Localization of Tu and aPC on different atlas sections (left). Localization from bregma is indicated for each section. Tetrode array tracks are shown in a coronal brain section (right).
d, Performance curves of the mouse cohort used in electrophysiology experiments (solid lines: mean, dashed lines: median, n = 88 sessions in 11 animals). Only mice were included that performed above criterion.
e, Average lick rate ± SEM during different trial types (n = 88 sessions in 11 animals).
f, Lick rate in the waiting window differentiated the respective trial types with CS100>CS50>CS0 (n = 88 sessions in 11 animals; one-way ANOVA with Tukey post-hoc comparisons). One-way ANOVA, $F(2,261) = 514.7$, $p = 2.6$ e-91, with Tukey post hoc comparison, p(CS0 vs CS50) = 9.6 e-10, p(CS0 vs CS100) = 9.6 e-10, p(CS50 vs CS100) = 9.6 e-10, * indicates $p < 0.05$.
g, Quality metrics to evaluate spike sorting. Action potential waveforms of an example single unit (left). Only units with less than 2% violations of the refractory spike period (middle) were included. Spike autocorrelation (right).
h, Baseline firing rate (left) and full width at half maximum (fwhm) of the action potential (right) of the analyzed single units (n = 169 units in Tu and n = 486 units in aPC).
Box plots in the figure: The bounds of the box represent 25th to 75th percentiles. The center indicates the median. The lower and upper whiskers represent the minimum and maximum values, excluding outliers. Source data are provided as a Source Data file.

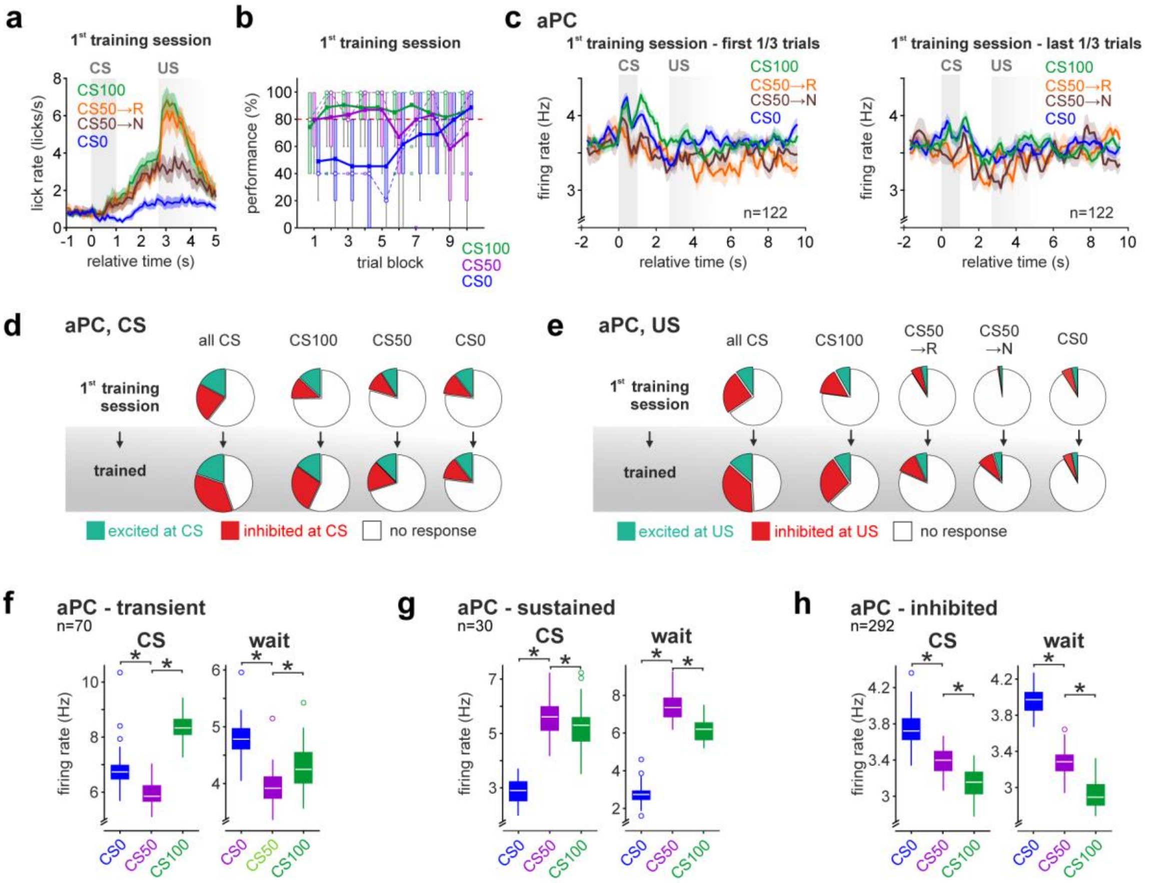

Supplementary Figure 7. Evolution of aPC task-related responses during training. Related to Figure 6.

a, Average licking rate ± SEM split by trial types for the first training sessions (n = 11 sessions in 11 animals).

b, Percentages of the correct CS100, CS50 and CS0 trials in first training sessions (n = 11 sessions in 11 animals). Solid lines indicate average performance while dashed lines indicate median performance.

c, Mean firing rate ± SEM of aPC units recorded during the first training sessions. Displayed mean firing rate during the first 50 trials (left) and the last 50 trials (right) of the session. Note that in the first trials of the first training session, mean aPC responses to the three odors partially differed at CS and during wait (see Supplementary Table 1 for exact p-values and test details), possibly reflecting intrinsic properties of the odors.

d-e, The fraction of task-inhibited responses at (d) CS and (e) US increased in aPC with training.

f-h, Box plots with median firing rate of the aPC (f) transient-cluster with n = 70 units, (g) sustained-cluster with n = 30 units and (h) inhibited-cluster with n = 292 units during CS and waiting period (one-way ANOVA with Tukey post-hoc comparisons).

In the figure: * indicates $p < 0.05$ (see Supplementary Table 1 for exact p-values and test details) and n indicates the number of units. Box plots: The bounds of the box represent 25th to 75th percentiles. The center indicates the median. The lower and upper whiskers represent the minimum and maximum values, excluding outliers. Source data are provided as a Source Data file.

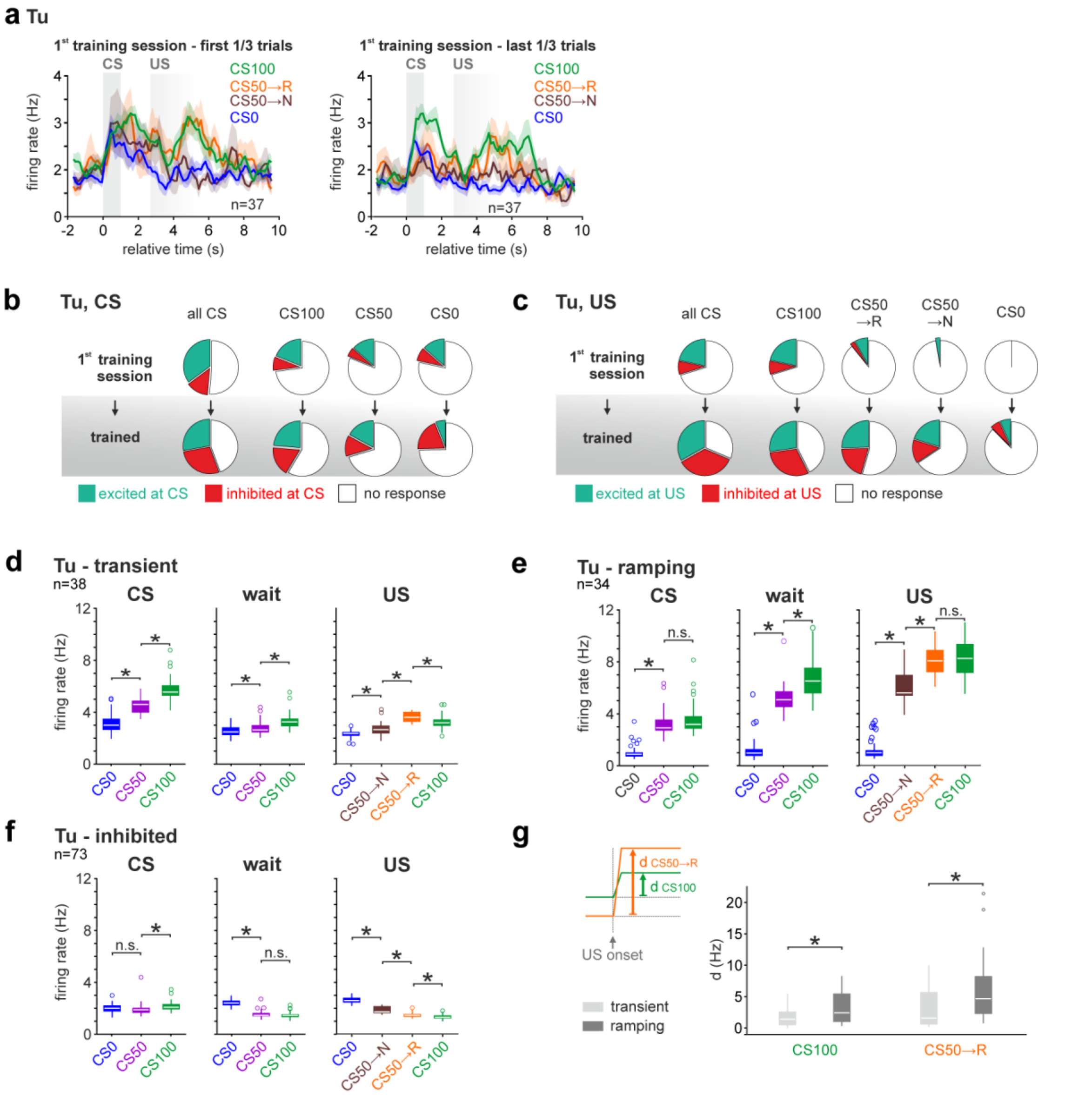

Supplementary Figure 8. Tu units form transient and ramping task response clusters. Related to Figure 7.

a, Same as Supplementary Fig. 7c for Tu. By the end of the first session, the Tu firing rate started reflecting RP coding by responding more strongly to CS100 than CS50 and CS0. Interestingly, at this stage, ramping activity is not yet seen during the waiting period. Note that while aPC encodes at CS the odors with responses of different intensities already at the beginning of the training phase (Supplementary Fig. 7c), this is not the case for Tu (see Supplementary Table 1 for exact p-values and test details).

b-c, Same as Supplementary Fig. 7d-e for Tu. Also in Tu, the fraction of task-inhibited responses increased with training for all trial types.

d-f, Same as Supplementary Fig. 7f-h for the Tu (d) transient-cluster with n = 38 units, (e) ramping-cluster with n = 34 units and (f) inhibited-cluster with n = 73 units during CS, waiting period, and US (one-way ANOVA with Tukey post-hoc comparisons).

g, Average reward response ± SEM of units with positive response to US from the transient- and ramping-cluster during the CS100 and the rewarded CS50 trials (n = 30 (CS100) and 30 (CS50→R) out of 38 transient units and n = 27 (CS100) and 30 (CS50→R) out of 34 ramping units). Reward response was computed as rate difference after US for each CS. Only units with positive average US response were included. Units from the ramping-cluster had a stronger reward response than those in the transient-cluster regardless of the certainty or not of the reward (CS100 or CS50, respectively) (two-tailed unpaired Wilcoxon rank-sum test).

In the figure: * indicates $p < 0.05$ (see Supplementary Table 1 for exact p-values and test details) and n indicates the number of units. Box plots: The bounds of the box represent 25th to 75th percentiles. The center indicates the median. The lower and upper whiskers represent the minimum and maximum values, excluding outliers. Source data are provided as a Source Data file.

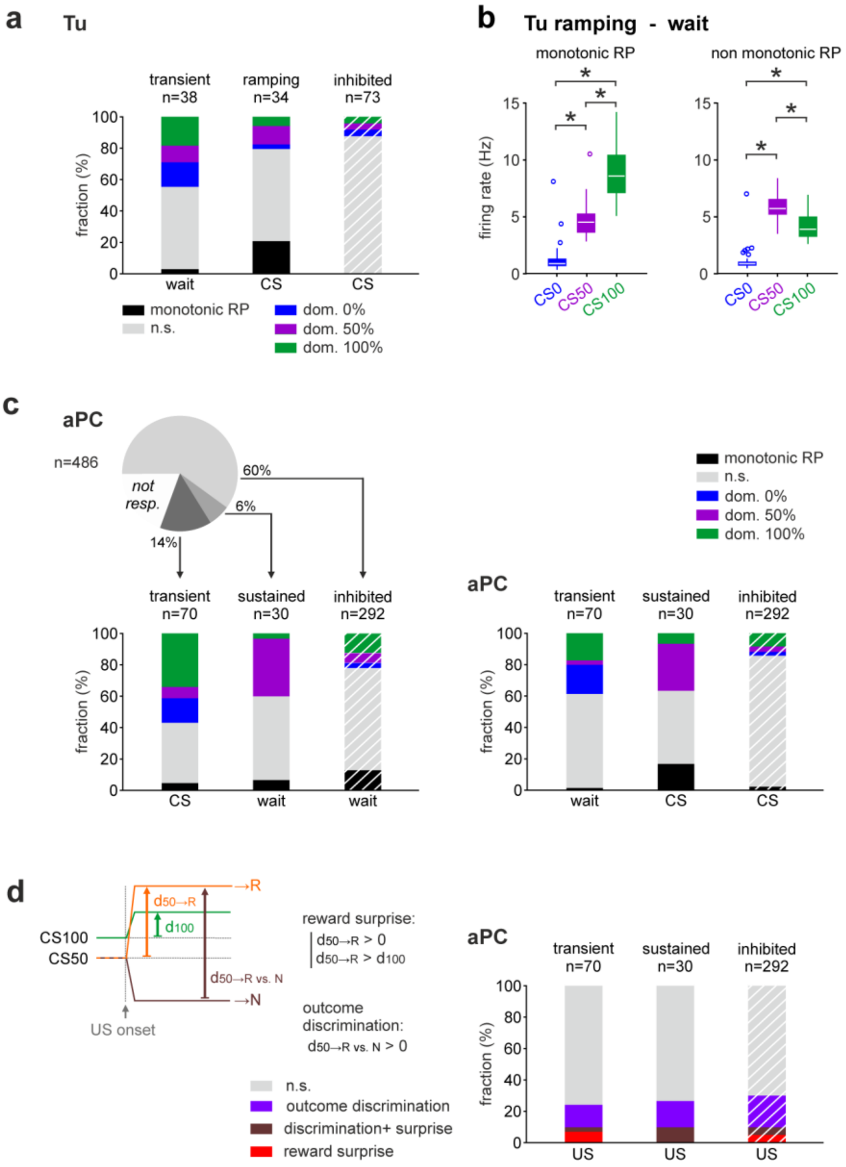

Supplementary Figure 9. Transient and ramping units differently encode monotonic RP. Related to Figure 8.
a, Same as Fig. 8a (bottom) but with complementary test windows. Note that coding in the three clusters changed between the CS and the waiting period.
b, Same as Fig. 8c but for the ramping Tu cluster group (left, n = 19 units; right, n = 15 units). No distributed monotonic RP coding was found in the Tu ramping group during the waiting period. * indicates $p < 0.05$ (see Supplementary Table 1 for exact p-values and test details). Box plots: The bounds of the box represent 25th to 75th percentiles. The center indicates the median. The lower and upper whiskers represent the minimum and maximum values, excluding outliers. Source data are provided as a Source Data file.
c, Same as Fig. 8a and (a) for aPC. The percentage of units in the three clusters defined in Fig. 6h-j coding individually for monotonic RP or showing a dominant activation for one of the three CS. Right: Complementary test windows. In aPC, only a small fraction of units in the inhibited-cluster encoded monotonic RP during the waiting period (in the task-excited clusters such fraction was below chance).
d, Same as Fig. 8d for aPC. Fraction of aPC units in the three clusters defined in Fig. 6h-j encoding reward surprise, outcome discrimination, or both. Note that units from all clusters contributed to some extent to PE coding.
In the figure: n indicates the number of units.

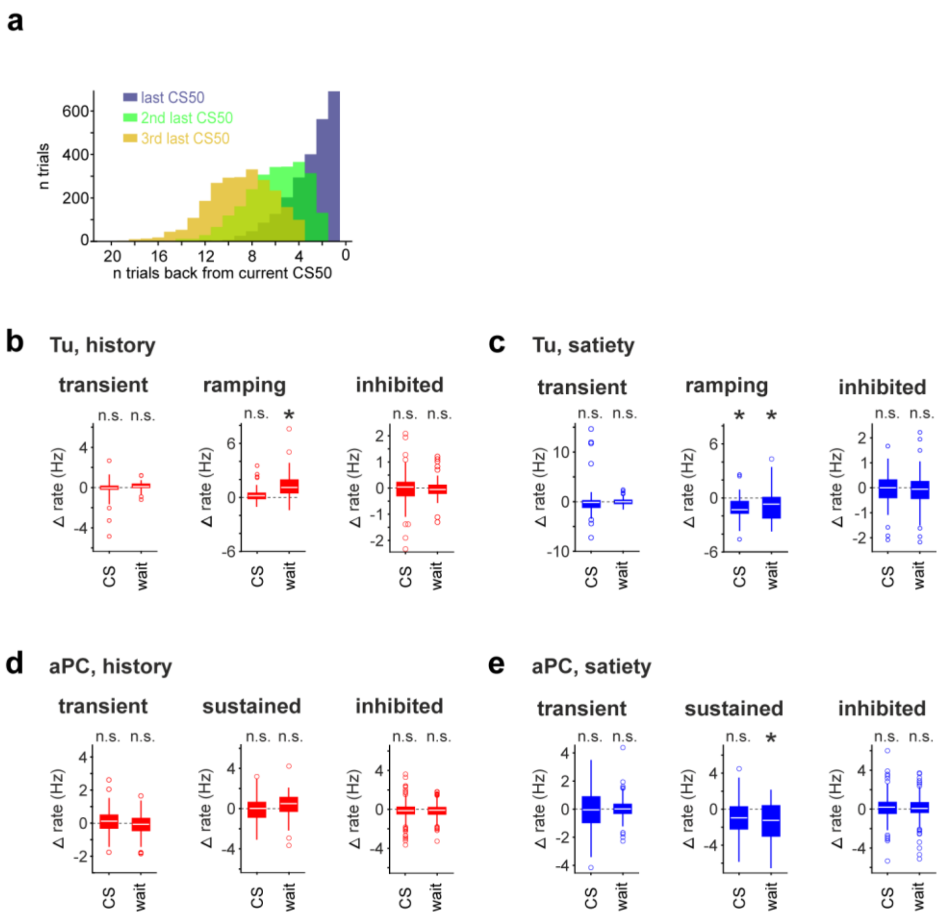

Supplementary Figure 10. Reward prediction updating through the recent cue-specific outcome-history. Related to Figure 9 and 10.

a, Histograms showing the distribution of the trial-gap between a CS50 trial and the respective last, second to last, and third to last CS50 trials, in all recording sessions (n = 88 sessions in 11 animals).

b-c, Difference in mean activity for the three major clusters in Tu for (b) outcome-history and (c) satiety (n = 38 transient-cluster units, n = 34 ramping-cluster units, n = 73 inhibited-cluster units). One-way repeated measures ANOVA with Greenhouse-Geisser and Bonferroni correction performed for history and satiety separately at CS and during waiting. Note that the ramping-cluster encoded both outcome-history and satiety during waiting.

d-e, Same as (b-c) for the aPC clusters (n = 70 transient-cluster units, n = 30 sustained-cluster units, n = 292 inhibited-cluster units). No cluster encoded outcome-history in aPC.

In the figure: * indicates $p < 0.05$ (see Supplementary Table 1 for exact p-values and test details). Box plots: The bounds of the box represent 25th to 75th percentiles. The center indicates the median. The lower and upper whiskers represent the minimum and maximum values, excluding outliers. Source data are provided as a Source Data file.

SUPPLEMENTARY TABLE

Supplementary Table 1. Statistical tests. Description and results of the statistical tests displayed in the manuscript figures. The first column of the table indicates the figure number and panel of the corresponding figure.

FIGURE	SAMPLE SIZE	STATISTICAL TEST	VALUES
**1c, wait**	[69, 69, 69]	One-way ANOVA with Tukey post hoc comparison	F(2,204) = 80.3, p = 1.9 e-26; post hoc comparisons: p(CS0 vs CS50) = 9.6 e-10, p(CS0 vs CS100) = 9.6 e-10, p(CS50 vs CS100) = 5.6 e-3
**1e, wait**	[10, 10, 10]	One-way ANOVA with Tukey post hoc comparison	F(2,27) = 21.2, p = 2.9 e-6; post hoc comparisons: p(CS0 vs CS50) = 3 e-3, p(CS0 vs CS100) = 1.7 e-6, p(CS50 vs CS100) = 0.02
**2e, history**	[10, 10]	two-tailed paired t-test	t(9) = 3.5, p = 7.3 e-3
**S1i, history**	[51, 51]	two-sided paired Wilcoxon signed rank test	Z = 6.2, p = 5.2 e-10
**5d, CS (left)**	[25, 25, 25]	One-way ANOVA with Tukey post hoc comparison	F(2,72) = 126.5, p = 2.7 e-24; post hoc comparisons: p(CS0 vs CS50) = 9.6 e-10, p(CS0 vs CS100) = 9.6 e-10, p(CS50 vs CS100) = 1.6 e-8
**5d, wait (right)**	[25, 25, 25]	One-way ANOVA with Tukey post hoc comparison	F(2,72) = 113.8, p = 5.2 e-23; post hoc comparisons: p(CS0 vs CS50) = 9.6 e-10, p(CS0 vs CS100) = 9.6 e-10, p(CS50 vs CS100) = 5.7 e-5
**5h, CS (left)**	[25, 25, 25]	One-way ANOVA with Tukey post hoc comparison	F(2,72) = 196.2, p = 7.2 e-30; post hoc comparisons: p(CS0 vs CS50) = 3.7 e-8, p(CS0 vs CS100) = 9.6 e-10, p(CS50 vs CS100) = 9.6 e-10
**5h, wait (right)**	[25, 25, 25]	One-way ANOVA	F(2,72) = 0.8, p = 0.5
**S6f, wait**	[88, 88, 88]	One-way ANOVA with Tukey post hoc comparison	F(2,261) = 514.7, p = 2.6 e-91; post hoc comparisons: p(CS0 vs CS50) = 9.6 e-10, p(CS0 vs CS100) = 9.6 e-10, p(CS50 vs CS100) = 9.6 e-10
**6b, CS (center)**	[50, 50, 50]	One-way ANOVA with Tukey post hoc comparison	F(2,147) = 17.7, p = 1.3 e-7; post hoc comparisons: p(CS0 vs CS50) = 1.0 e-6, p(CS0 vs CS100) = 1.0, p(CS50 vs CS100) = 6.2 e-7
**6b, wait (right)**	[50, 50, 50]	One-way ANOVA with Tukey post hoc comparison	F(2,147) = 189.0, p = 2.3 e-41; post hoc comparisons: p(CS0 vs CS50) = 9.6 e-10, p(CS0 vs CS100) = 9.6 e-10, p(CS50 vs CS100) = 9.6 e-10
**S7c, CS (left)**	[17, 14, 17]	One-way ANOVA with Tukey post hoc comparison	F(2,45) = 20.2, p = 5.4 e-7; post hoc: p(CS0 vs CS50) = 2.8 e-6, p(CS0 vs CS100) = 9.9 e-1, p(CS50 vs CS100) = 4.8 e-6
**S7c, wait (left)**	[17, 14, 17]	One-way ANOVA with Tukey post hoc comparison	F(2,45) = 23.1, p = 1.3 e-7; post hoc: p(CS0 vs CS50) = 1.8 e-3, p(CS0 vs CS100) = 5.5 e-3, p(CS100,CS50) = 6.0 e-8
**S7f, CS (left)**	[50, 50, 50]	One-way ANOVA with Tukey post hoc comparison	F(2,147) = 242.4, p = 2.9 e-47; post hoc comparisons: p(CS0 vs CS50) = 9.6 e-10, p(CS0 vs CS100) = 9.6 e-10, p(CS50 vs CS100) = 9.6 e-10
**S7f, wait (right)**	[50, 50, 50]	One-way ANOVA with Tukey post hoc comparison	F(2,147) = 88.0, p = 7.5 e-26; post hoc comparisons: p(CS0 vs CS50) = 9.6 e-10, p(CS0 vs CS100) = 9.6 e-10, p(CS50 vs CS100) = 1.2 e-7
**S7g, CS (left)**	[50, 50, 50]	One-way ANOVA with Tukey post hoc comparison	F(2,147) = 287.0, p = 1.7 e-51; post hoc comparisons: p(CS0 vs CS50) = 9.6 e-10, p(CS0 vs CS100) = 9.6 e-10, p(CS50 vs CS100) = 0.048
**S7g, wait (right)**	[50, 50, 50]	One-way ANOVA with Tukey post hoc comparison	F(2,147) = 764.6, p = 2.0 e-78; post hoc comparisons: p(CS0 vs CS50) = 9.6 e-10, p(CS0 vs CS100) = 9.6 e-10, p(CS50 vs CS100) = 1.1 e-9

**S7h, CS (left)**	[50, 50, 50]	One-way ANOVA with Tukey post hoc comparison	F(2,147) = 156.7, p = 3.6 e-37; post hoc comparisons: p(CS0 vs CS50) = 9.6 e-10, p(CS0 vs CS100) = 9.6 e-10, p(CS50 vs CS100) = 9.6 e-10
**S7h, wait (right)**	[50, 50, 50]	One-way ANOVA with Tukey post hoc comparison	F(2,147) = 521.6, p = 1.7 e-67; post hoc comparisons: p(CS0 vs CS50) = 9.6 e-10, p(CS0 vs CS100) = 9.6 e-10, p(CS50 vs CS100) = 9.6 e-10
**7b, CS (center)**	[50, 50, 50]	One-way ANOVA with Tukey post hoc comparison	F(2,139) = 141.1, p = 3.5 e-34; post hoc comparisons: p(CS0 vs CS50) = 9.6 e-10, p(CS0 vs CS100) = 9.6 e-10, p(CS50 vs CS100) = 1.0 e-7
**7b, wait (right)**	[50, 50, 50]	One-way ANOVA with Tukey post hoc comparison	F(2,139) = 99.0, p = 1.8 e-27; post hoc comparisons: p(CS0 vs CS50) = 9.6 e-10, p(CS0 vs CS100) = 9.6 e-10, p(CS50 vs CS100) = 3.9 e-7
**S8a, CS (left)**	[17, 14, 17]	One-way ANOVA	F(2,45) = 1.0, p = 3.7 e-01
**S8a, wait (left)**	[17, 14, 17]	One-way ANOVA with Tukey post hoc comparison	F(2,45) = 6.3, p = 4.0 e-3; post hoc: p(CS0 vs CS50) = 1.0, p(CS0 vs CS100) = 1.2 e-2, p(CS100 vs CS50) = 9.9 e-3
**S8d, CS (left)**	[50, 50, 50]	One-way ANOVA with Tukey post hoc comparison	F(2,147) = 168.7 p = 8.7 e-39; post hoc comparisons: p(CS0 vs CS50) = 9.6 e-10, p(CS0 vs CS100) = 9.6 e-10, p(CS50 vs CS100) = 9.6 e-10
**S8d, wait (center)**	[50, 50, 50]	One-way ANOVA with Tukey post hoc comparison	F(2,139) = 37.4, p = 7.3 e-14; post hoc comparisons: p(CS0 vs CS50) = 5.0 e-3, p(CS0 vs CS100) = 9.6 e-10, p(CS50 vs CS100) = 1.8 e-7
**S8d, US (right)**	[50,21,21,50]	One-way ANOVA with Tukey post hoc comparison	F(3,138) = 70.5, p = 1.0 e-27; post hoc comparisons: p(CS0 vs CS50N) =3.4 e-4, p(CS0 vs CS50R) = 3.8 e-9, p(CS0 vs CS100) = 3.8 e-9, p(CS50N vs CS50R) = 3.8 e-9, p(CS50N vs CS100) = 1.8 e-5, p(CS50R vs CS100) = 5.0 e-4
**S8e, CS (left)**	[50, 50, 50]	One-way ANOVA with Tukey post hoc comparison	F(2,147) = 281.1, p = 5.9 e-51; post hoc comparisons: p(CS0 vs CS50) = 9.6 e-10, p(CS0 vs CS100) = 9.6 e-10, p(CS50 vs CS100) = 0.4
**S8e, wait (center)**	[50, 50, 50]	One-way ANOVA with Tukey post hoc comparison	F(2,147) = 486.7, p = 1.5 e-65; post hoc comparisons: p(CS0 vs CS50) = 9.6 e-10, p(CS0 vs CS100) = 9.6 e-10, p(CS50 vs CS100) = 6.0 e-4
**S8e, US (right)**	[50,21,21,50]	One-way ANOVA with Tukey post hoc comparison	F(3,138) = 488.4, p = 2.9 e-73; post hoc comparisons: p(CS0 vs CS50N) = 3.8 e-9, p(CS0 vs CS50R) = 3.8 e-9, p(CS0 vs CS100) = 3.8 e-9, p(CS50N vs CS50R) = 6.8 e-6, p(CS50N vs CS100) = 8.8 e-9, p(CS50R vs CS100) = 1.0
**S8f, CS (left)**	[50, 50, 50]	One-way ANOVA with Tukey post hoc comparison	F(2,147) = 7.7, p = 6.9 e-4; post hoc comparisons: p(CS0 vs CS50) = 0.5, p(CS0 vs CS100) = 0.02, p(CS50 vs CS100) = 4.2 e-4
**S8f, wait (center)**	[50, 50, 50]	One-way ANOVA with Tukey post hoc comparison	F(2,147) = 193.7, p = 6.3 e-42; post hoc comparisons: p(CS0 vs CS50) = 9.6 e-10, p(CS0 vs CS100) = 9.6 e-10, p(CS50 vs CS100) = 0.3
**S8f, US (right)**	[50,21,21,50]	One-way ANOVA with Tukey post hoc comparison	F(3,138) = 277.2, p = 3.3 e-58; post hoc comparisons: p(CS0 vs CS50N) = 3.8 e-9, p(CS0 vs CS50R) = 3.8 e-9, p(CS0 vs CS100) = 3.8 e-9, p(CS50N vs CS50R) = 3.9 e-8, p(CS50N vs CS100) = 3.8 e-9, p(CS50R vs CS100) = 0.02
**S8g left**	[30,27]	Two-tailed unpaired Wilcoxon rank-sum test	Z = -2.7, p = 0.007
**S8g right**	[30,30]	Two-tailed unpaired Wilcoxon rank-sum test	Z = -2.9, p = 0.004

**8c, monotonic (left)**	[50, 50, 50]	One-way ANOVA with Tukey post hoc comparison	F(2,147) = 109.6, p = 7.2 e-30; post hoc comparisons: p(CS0 vs CS50) = 9.6 e-10, p(CS0 vs CS100) = 9.6 e-10, p(CS50 vs CS100) = 1.0 e-9
**8c, non-monotonic (right)**	[50, 50, 50]	One-way ANOVA with Tukey post hoc comparison	F(2,147) = 92.0, p = 1.2 e-26; post hoc comparisons: p(CS0 vs CS50) = 9.6 e-10, p(CS0 vs CS100) = 9.6 e-10, p(CS50 vs CS100) = 4.7 e-6
**S9b, monotonic (left)**	[50, 50, 50]	One-way ANOVA with Tukey post hoc comparison	F(2,147) = 361.4, p = 1.8 e-57; post hoc comparisons: p(CS0 vs CS50) = 9.6 e-10, p(CS0 vs CS100) = 9.6 e-10, p(CS50 vs CS100) = 9.6 e-10
**S9b, non-monotonic (right)**	[50, 50, 50]	One-way ANOVA with Tukey post hoc comparison	F(2,147) = 380.8, p = 7.2 e-59; post hoc comparisons: p(CS0 vs CS50) = 9.6 e-10, p(CS0 vs CS100) = 9.6 e-10, p(CS50 vs CS100) = 9.6 e-10
**9c left**	[88, 88]	two-tailed paired t-test	t(87) = 8.2, p = 2.4 e-12
**9c right**	[88, 88]	two-tailed paired t-test	t(87) = 7.5, p = 4.6 e-11
**9d, history**	[88, 88]	two-tailed paired t-test	t(87) = 12.3, p = 0.0
**9e**	[88]<br>[88]<br>[88]<br>[88]<br>[88]<br>[88]	two-tailed one-sample t-test	n-1: t(87) = 8.8; p = 1.2 e-13<br>n-2: t(87) = 6.2; p = 1.7 e-9<br>n-3: t(87) = 4.1; p = 1 e-4<br>n-4: t(87) = 2.5; p = 0.01<br>n-5: t(87) = 1.7; p = 0.08<br>n-6: t(87) = 0.2; p = 0.88
**9f**	[88]<br>[88]<br>[88]<br>[88]<br>[88]<br>[88]	two-tailed one-sample t-test	n-1: t(87) = -6.2; p = 2.1 e-8<br>n-2: t(87) = -3.9; p = 1.9 e-4<br>n-3: t(87) = -1.6; p = 0.10<br>n-4: t(87) = -1.5; p = 0.13<br>n-5: t(87) = -0.2; p = 0.87<br>n-6: t(87) = 0.7; p = 0.48
**10a, left<br>10b, left<br>(CS)**	[10, 10, 6, 6]	Joined history and satiety test at *CS*:<br>One-way ANOVA	F(3,28) = 0.3, p = 0.8
**10a, left<br>10b, left<br>(wait)**	[10, 10, 6, 6]	Joined history and satiety test during *wait*:<br>One-way ANOVA with Tukey post hoc comparison	F(3,28) = 10.9, p = 6.4 e-5;<br>post hoc comparisons: p(history) = 7.1 e-5, p(satiety) = 0.4
**10a, right<br>10b, right<br>(CS)**	[10, 10, 6, 6]	Joined history and satiety test at *CS*:<br>One-way ANOVA with Tukey post hoc comparison	F(3,28) = 8.4, p = 3.7 e-4;<br>post hoc comparisons: p(history) = 0.6 p(satiety) = 3.1 e-4
**10a, right<br>10b, right<br>(wait)**	[10, 10, 6, 6]	Joined history and satiety test during *wait*:<br>One-way ANOVA with Tukey post hoc comparison	F(3,28) = 4.4, p = 1.2 e-2;<br>post hoc comparisons: p(history) = 0.5 p(satiety) = 0.01
**10c, left**		see statistic S10B,S10C	
**10c, right**		see statistic S10B,S10C	
**S10b, left<br>S10c, left<br>(CS)**	[38,38,38,38]	Joined history and satiety test at *CS*:<br>One-way repeated measures ANOVA with Greenhouse-Geisser and Bonferroni correction	F(1.2,44.1) = 0.2, p = 0.7
**S10b, left<br>S10c, left<br>(wait)**	[38,38,38,38]	Joined history and satiety test during *wait*:	F(1.6,59.7) = 0.9, p = 0.4

		One-way repeated measures ANOVA with Greenhouse-Geisser and Bonferroni correction	
**S10b, middle S10c, middle (CS)**	[34,34,34,34]	Joined history and satiety test at *CS*:<br>One-way repeated measures ANOVA with Greenhouse-Geisser and Bonferroni correction	F(1.9,62.1) = 16.9, p = 2.0 e-6;<br>post hoc comparisons: p(history) =0.2, p(satiety) = 1.8 e-4
**S10b, middle S10c, middle (wait)**	[34,34,34,34]	Joined history and satiety test during *wait*:<br>One-way repeated measures ANOVA with Greenhouse-Geisser and Bonferroni correction	F(2.3,75.3) = 20.9, p = 1.4 e-8<br>post hoc comparisons: p(history) = 2.7 e-7, p(satiety) = 0.02
**S10b, right S10c, right (CS)**	[73, 73, 73, 73]	Joined history and satiety test at *CS*:<br>One-way repeated measures ANOVA with Greenhouse-Geisser and Bonferroni correction	F(2.5,183.0) = 0.01, p = 1.0
**S10b, right S10c, right (wait)**	[73, 73, 73, 73]	Joined history and satiety test during *wait*:<br>One-way repeated measures ANOVA with Greenhouse-Geisser and Bonferroni correction	F(2.0,145.1) = 0.4, p = 0.6
**S10d, left S10e, left (CS)**	[70, 70, 70, 70]	Joined history and satiety test at *CS*:<br>One-way repeated measures ANOVA with Greenhouse-Geisser and Bonferroni correction	F(1.8, 126.0) = 0.8, p = 0.4
**S10d, left S10e, left (wait)**	[70, 70, 70, 70]	Joined history and satiety test during *wait*:<br>One-way repeated measures ANOVA with Greenhouse-Geisser and Bonferroni correction	F(2.3,162.0) = 0.9, p = 0.4
**S10d, middle S10e, middle (CS)**	[30, 30, 30, 30]	Joined history and satiety test at *CS*:<br>One-way repeated measures with Greenhouse-Geisser and Bonferroni correction	F(1.7,48.8) = 4.5, p = 0.02;<br>post hoc comparisons: p(history) = 1.0, p(satiety) = 0.1
**S10d, middle S10e, middle (wait)**	[30, 30, 30, 30]	Joined history and satiety test during *wait*:<br>One-way repeated measures ANOVA with Greenhouse-Geisser and Bonferroni correction	F(1.8,52.6) = 7.7, p = 0.002;<br>post hoc comparisons: p(history) = 1.0, p(satiety) = 0.02
**S10d, right S10e, right (CS)**	[292, 292, 292, 292]	Joined history and satiety test at *CS*:<br>One-way repeated measures ANOVA with Greenhouse-Geisser and Bonferroni correction	F(2.2,646.9) = 5.3, p = 0.004;<br>post hoc comparisons: p(history) = 0.06, p(satiety) = 0.1;
**S10d, right S10e, right (wait)**	[292, 292, 292, 292]	Joined history and satiety test during *wait*:<br>One-way repeated measures ANOVA with Greenhouse-Geisser and Bonferroni correction	F(2.1,601.0) = 5.9, p = 0.003;<br>post hoc comparisons: p(history) = 0.2, p(satiety) = 0.06
