# Supplementary material for: Striatal hub of dynamic and stabilized prediction coding in forebrain networks for olfactory reinforcement learning
Source: Nat Commun. 2022 Jun 8;13:3305. doi: 10.1038/s41467-022-30978-1 (PMC9177857; doi:10.1038/s41467-022-30978-1)
Supplement: Supplementary file 3 — Reporting Summary [file 41467_2022_30978_MOESM3_ESM.pdf]

nature research

Corresponding author(s): Wolfgang Kelsch

Last updated by author(s): Apr 24, 2022

Reporting Summary

Nature Research wishes to improve the reproducibility of the work that we publish. This form provides structure for consistency and transparency in reporting. For further information on Nature Research policies, see Authors & Referees and the Editorial Policy Checklist .

Statistics

For all statistical analyses, confirm that the following items are present in the figure legend, table legend, main text, or Methods section.

n/a	Confirmed	
☐	☒	The exact sample size (*n*) for each experimental group/condition, given as a discrete number and unit of measurement
☐	☒	A statement on whether measurements were taken from distinct samples or whether the same sample was measured repeatedly
☐	☒	The statistical test(s) used AND whether they are one- or two-sided<br>*Only common tests should be described solely by name; describe more complex techniques in the Methods section.*
☐	☒	A description of all covariates tested
☐	☒	A description of any assumptions or corrections, such as tests of normality and adjustment for multiple comparisons
☐	☒	A full description of the statistical parameters including central tendency (e.g. means) or other basic estimates (e.g. regression coefficient) AND variation (e.g. standard deviation) or associated estimates of uncertainty (e.g. confidence intervals)
☐	☒	For null hypothesis testing, the test statistic (e.g. *F*, *t*, *r*) with confidence intervals, effect sizes, degrees of freedom and *P* value noted<br>*Give P values as exact values whenever suitable.*
☒	☐	For Bayesian analysis, information on the choice of priors and Markov chain Monte Carlo settings
☐	☒	For hierarchical and complex designs, identification of the appropriate level for tests and full reporting of outcomes
☐	☒	Estimates of effect sizes (e.g. Cohen's *d*, Pearson's *r*), indicating how they were calculated

Our web collection on statistics for biologists contains articles on many of the points above.

Software and code

Policy information about availability of computer code

Data collection	- Matlab 2017b<br>- Arduino Software 1.8.5<br>- Intan Technologies Acquisition Software 1.4.2<br>- Bruker Paravision Software (Version 6)
Data analysis	- Matlab (2017a / 2019b)<br>- MLIB toolbox (Version 6)<br>- Statistical Parametric Mapping 12 (SPM12)<br>- fastICA package (Version 2.5)<br>- Sandwich Estimator Toolbox (Version 1.2.5)<br>- ArtRepair Toolbox (Version 5b)<br>- BASCO Toolbox (Version 2)<br>- IBM SPSS 22<br>- Corel Draw 2019

For manuscripts utilizing custom algorithms or software that are central to the research but not yet described in published literature, software must be made available to editors/reviewers. We strongly encourage code deposition in a community repository (e.g. GitHub). See the Nature Research guidelines for submitting code & software for further information.

Data

Policy information about availability of data

All manuscripts must include a data availability statement. This statement should provide the following information, where applicable:

- Accession codes, unique identifiers, or web links for publicly available datasets
- A list of figures that have associated raw data
- A description of any restrictions on data availability

The single unit recording data generated in this study are provided in the Supplementary Information/Source Data file.

The fMRI and electrophysiology data generated in this study are under active use by the reporting laboratory; all data presented in this manuscript are available by reasonable request (for MRI data: christian.clemm@zi-mannheim.de, for electrophysiology data: wokelsch@uni-mainz.de). Source data are provided with this paper. BOLD fMRI statistical maps and electrophysiological single unit spike counts are available for download. Matlab code and pipeline description for pupil data analyses and MRI brain mask creation are available at https://github.com/DrCarbonCIMH/extractPupil and https://github.com/DrCarbonCIMH/extractBrain, respectively.

Field-specific reporting

Please select the one below that is the best fit for your research. If you are not sure, read the appropriate sections before making your selection.

☒ Life sciences ☐ Behavioural & social sciences ☐ Ecological, evolutionary & environmental sciences

For a reference copy of the document with all sections, see nature.com/documents/nr-reporting-summary-flat.pdf

Life sciences study design

All studies must disclose on these points even when the disclosure is negative.

Sample size	Sample sizes (number of single units within a single analyses and number of animals per group) were chosen according to previously used numbers in published awake recordings (eg. Oettl et al. (2020), Nat. Comm. 11(1):3460) sufficient to detect medium or large size effects. Since the exact variability of the effects was unknown, sample size calculations were not reliably possible.
Data exclusions	For both fMRI and cellular unit recordings, only behavioral sessions were included in which the animal reached criterion (80% correct (hit and correct rejection) trials).<br><br>Single-units were only included in the further analyses if their fraction of spikes violating the refractory period was less than 2 %. To select putative striatal projection neurons in the olfactory tubercle, we restricted to units with a baseline firing rate between 0 Hz and 5 Hz. In the anterior piriform cortex, only units with a baseline firing rate below 10 Hz were considered.
Replication	The study examined a novel phenomenon. The study design did not contain generally full replication cohorts, however, analyses were designed to test the presence of the described phenomenon across animals in the fMRI (group-level inference, taking into account repeated measures unbalanced between animals).
Randomization	Mouse cohorts for fMRI and cellular recordings were of the same sex, genotype and comparable age (ca. 12 weeks at the beginning of the experiment). All animals underwent the same experimental procedure. Therefore, in both experiments, no further randomizations were necessary at the subject level.<br>Trial sequences within a session: three distinct odors delivered in a pseudo-random order to keep the proportion of odors constant between sessions. No stimulus was consecutively applied more than three times in a row. No more than three consecutive trials were rewarded. Further, the trial duration was randomly drawn from a uniform distribution between 10 and 12 s.
Blinding	In the experiments, blinding was not relevant as all animals received the same treatment (training, task performance and analyses).

Reporting for specific materials, systems and methods

We require information from authors about some types of materials, experimental systems and methods used in many studies. Here, indicate whether each material, system or method listed is relevant to your study. If you are not sure if a list item applies to your research, read the appropriate section before selecting a response.

Materials & experimental systems

n/a	Involved in the study
☒	☐ Antibodies
☒	☐ Eukaryotic cell lines
☒	☐ Palaeontology
☐	☒ Animals and other organisms
☒	☐ Human research participants
☒	☐ Clinical data

Methods

n/a	Involved in the study
☒	☐ ChIP-seq
☒	☐ Flow cytometry
☐	☒ MRI-based neuroimaging

Animals and other organisms

Policy information about studies involving animals; ARRIVE guidelines recommended for reporting animal research

Laboratory animals	Ten week old, male C57BL/6N mice were obtained directly from Charles River Laboratories (23 animals for the fMRI measurements, 11 animals for single-unit recordings). Mice were housed individually in a standard 12 hours light-dark-cycle. Food and water were given ad libitum, except when water supply was controlled for behavioral training. Mice were single housed upon implantation of the recording array or head-bar at a 12 hours' day-and-night-cycle (room temperature 24°C, air humidity 55%). Mice were 3 to 6 months old when recordings and fMRI were performed.
Wild animals	No wild animals were used in the study.
Field-collected samples	No field collected samples were used in the study.
Ethics oversight	Referat 35, Regierungspraesidium Karlsruhe, State of Baden-Wuerttemberg, Germany

Note that full information on the approval of the study protocol must also be provided in the manuscript.

Magnetic resonance imaging

Experimental design

Design type	Odor-reward association learning task; event-related design
Design specifications	Three different stimulus-outcome pairs were presented in random order, corresponding to three different odors (CS) each with a specific reward probability. Each trial started with 1 s of odor presentation followed by a waiting period of 1.7 s. Reward (US) was delivered immediately after the waiting period. The trial duration was randomly drawn from a uniform distribution between 10 and 12 s. To increase statistical power, animals were repeatedly measured (up to 5 sessions per animal).
Behavioral performance measures	Licking behavior was measured during fMRI acquisition. Only behavioral sessions were included in which the animal reached criterion (80% correct (hit and rejection) trials). If mice licked at least three times during the anticipatory window (from 1.5 s to 2.8 s after odor onset) or in the reward window (from 2.8 s to 4.1 s), it was considered as a go-response. Fulfilling the lick criterion during 'Go' trials was regarded as a 'Hit', while it was assessed as 'False alarm' during 'No-go' trials. Not meeting the lick criterion was regarded as 'Correct rejection' for 'No-go' trials and as 'Miss' for 'Go' trials. Of the 67 completed scanning sessions, 51 sessions in 18 animals performed above criterion.<br><br>performance = (number of '"Hit' " trials + number of 'Correct rejection' trials)/(total number of trials)

Acquisition

Imaging type(s)	Functional Magnetic Resonance Imaging
Field strength	9.4 Tesla (MRI scanner: 94/20 Bruker Biospec, Ettlingen Germany)
Sequence & imaging parameters	The fMRI time series were acquired while mice performed the odor task using an echo-planar imaging gradient echo (EPI-FID) sequence with the following parameters: TR/TE: 1300/17 ms; flip angle: 50°; 21 slices; matrix size: 64 x 64; slice thickness: 0.5 mm; interslice gap: 0.1 mm; voxel size: 0.25 x 0.25 x 0.6 mm; 1400 volume acquisitions.
Area of acquisition	The area of acquisition comprised the forebrain excluding brainstem and cerebellum. More posterior slices frequently had ventral signal dropout due to B0 field inhomogeneity. Therefore only slices from +4.2 mm to 0 mm relative to Bregma (i.e. anteriorly comprising the olfactory bulb and spanning olfactory, striatal and higher-order cortical regions) were considered. Note, however, that multiple-comparison correction during statistical inference was always based on the voxels in all slices originally acquired.

Diffusion MRI ☐ Used ☒ Not used

Preprocessing

Preprocessing software	fMRI data were preprocessed using SPM12 with the following steps: discarding the first five volumes in the series to avoid influences of magnetization before the scanner achieves steady state, correction for head movement by realignment to the middle volume using a rigid-body transformation, correction for geometrical distortions using the acquired B0-field maps, slice-timing correction, and spatial normalization to a mouse brain template in the Paxinos stereotactic coordinate system, by applying the non-linear normalization parameters of the individual structural images to the functional images. Spatially normalized functional images were additionally smoothed with a 0.6 mm isotropic Gaussian kernel.
Normalization	BOLD data were spatially normalized to a population-averaged mouse brain template in the Paxinos stereotactic coordinate space by applying the non-linear normalization parameters of the structural images to the functional images.
Normalization template	Structural images were non-linearly normalized by segmentation to high-resolution tissue probability maps (Biedermann et al., 2012. In vivo voxel based morphometry: detection of increased hippocampal volume and decreased glutamate levels in exercising mice) transformed to Paxinos space.
Noise and artifact removal	We employed a combination of denoising methods to minimize effects of head-motion and physiological artifacts:<br>- Group-independent component analysis (ICA)<br>- Cerebrospinal fluid (CSF) signal and licking events were included as a nuissance regressor in the first-level general linear model
Volume censoring	We did not apply any form of volume censoring to our fMRI data.

Statistical modeling & inference

Model type and settings	Univariate analysis. 1st level: In SPM12, a general linear model (fixed effects) was used, where odor and reward time-points were modelled as events (stick functions) convolved with a mouse-specific HRF. As described in detail in the manuscript, events were parametrically modulated with quantitative parameters from the behavioral modelling. 2nd level: we used the Sandwich-Estimator Toolbox (Guillaume et al., 2014. Fast and accurate modelling of longitudinal and repeated measures neuroimaging data), which allows for unequal numbers of longitudinal measurements between subjects, and where random effects are accounted for using an unstructured error covariance.
Effect(s) tested	Using the Sandwich Estimator Toolbox as described above, we tested whether the following beta coefficients (or linear contrasts between beta coefficients) were significantly different from zero: parametric modulators (stimulus value, reward size, last outcome of ambiguous trial), CS100 vs. CS50, and CS50 vs. CS0. See manuscript for details.

Specify type of analysis: [x] Whole brain [ ] ROI-based [ ] Both

Statistic type for inference (See Eklund et al. 2016)	Voxel-wise statistical inference
Correction	FDR-correction

Models & analysis

n/a	Involved in the study
[ ]	[x] Functional and/or effective connectivity
[x]	[ ] Graph analysis
[x]	[ ] Multivariate modeling or predictive analysis

Functional and/or effective connectivity	The event-related functional (undirected) connectivity analysis employed a beta-series correlation method (Rissmann et al. 2014), utilizing the BASCO toolbox (Gottlich et al. 2015). Here, fMRI data were incorporated into another session-wise GLM, in which each event (CS and US) of each trial was modeled as a separate regressor to obtain the respective beta weights. For ROI-based analyses, betas were averaged over all voxels of a given region and the Pearson's correlation coefficient was computed between region pairs. The event-related ROI-based functional connectivity was computed across sessions for each event type (e.g. US time point of CS100 trial type). Using the same session-wise GLMs, a seed-based functional connectivity analysis was performed for two seed regions (i.e., one cortical, one subcortical). The beta series of each seed was correlated to that of each voxel contained within the odor-reward association learning network. A group-level analysis of the single session seed-based connectivity results was computed.
